# Supplementary material for: Glioblastoma Myeloid-Derived Suppressor Cell Subsets Express Differential Macrophage Migration Inhibitory Factor Receptor Profiles That Can Be Targeted to Reduce Immune Suppression
Source: Front Immunol. 2020 Jun 18;11:1191. doi: 10.3389/fimmu.2020.01191 (PMC7315581; doi:10.3389/fimmu.2020.01191)

Supplemental Figure 1. MIF knockdown reduced myeloid infiltration into tumors and does not increase survival in a mouse mode of GBM using immune incompetent mice

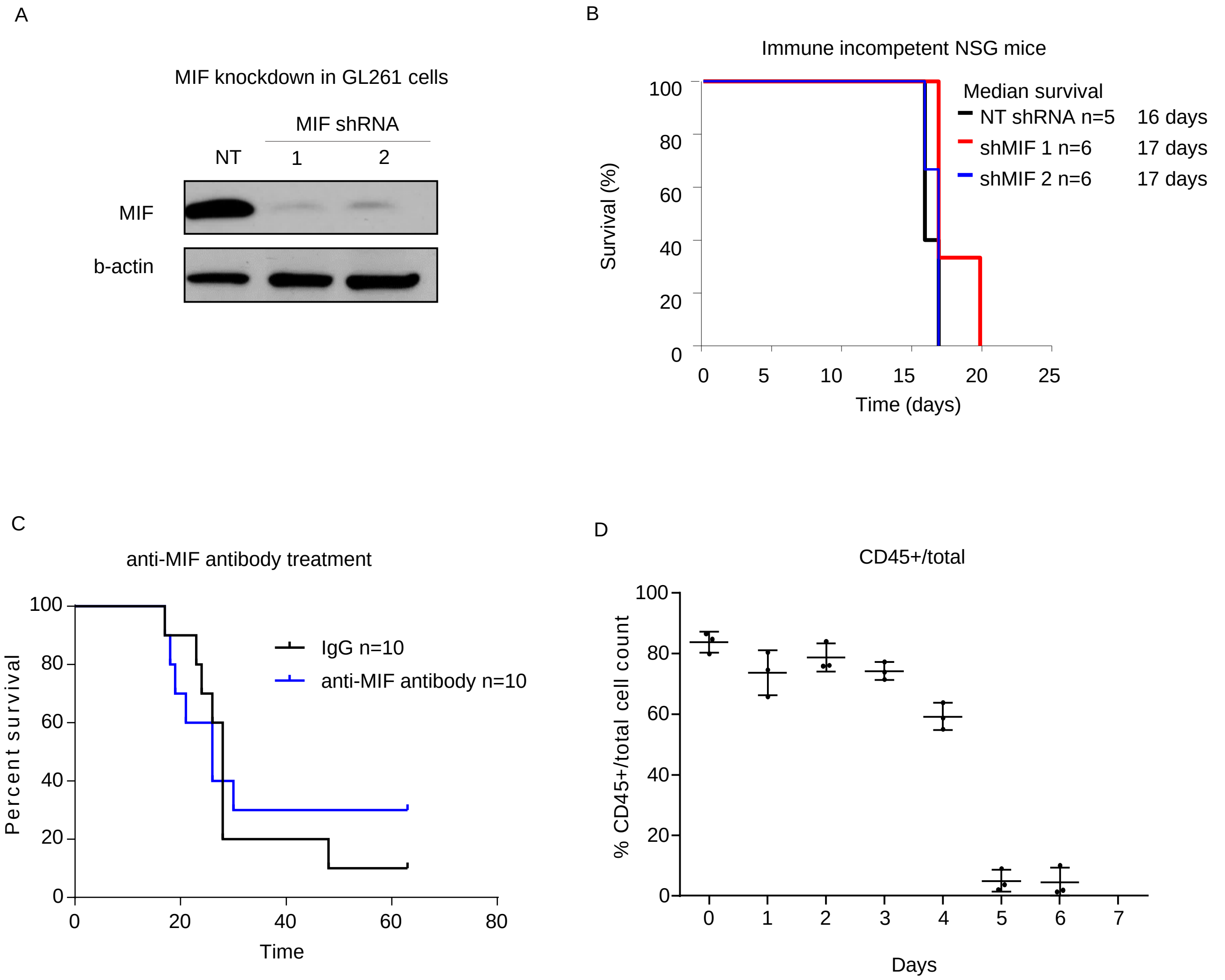

Supplemental Figure 2.

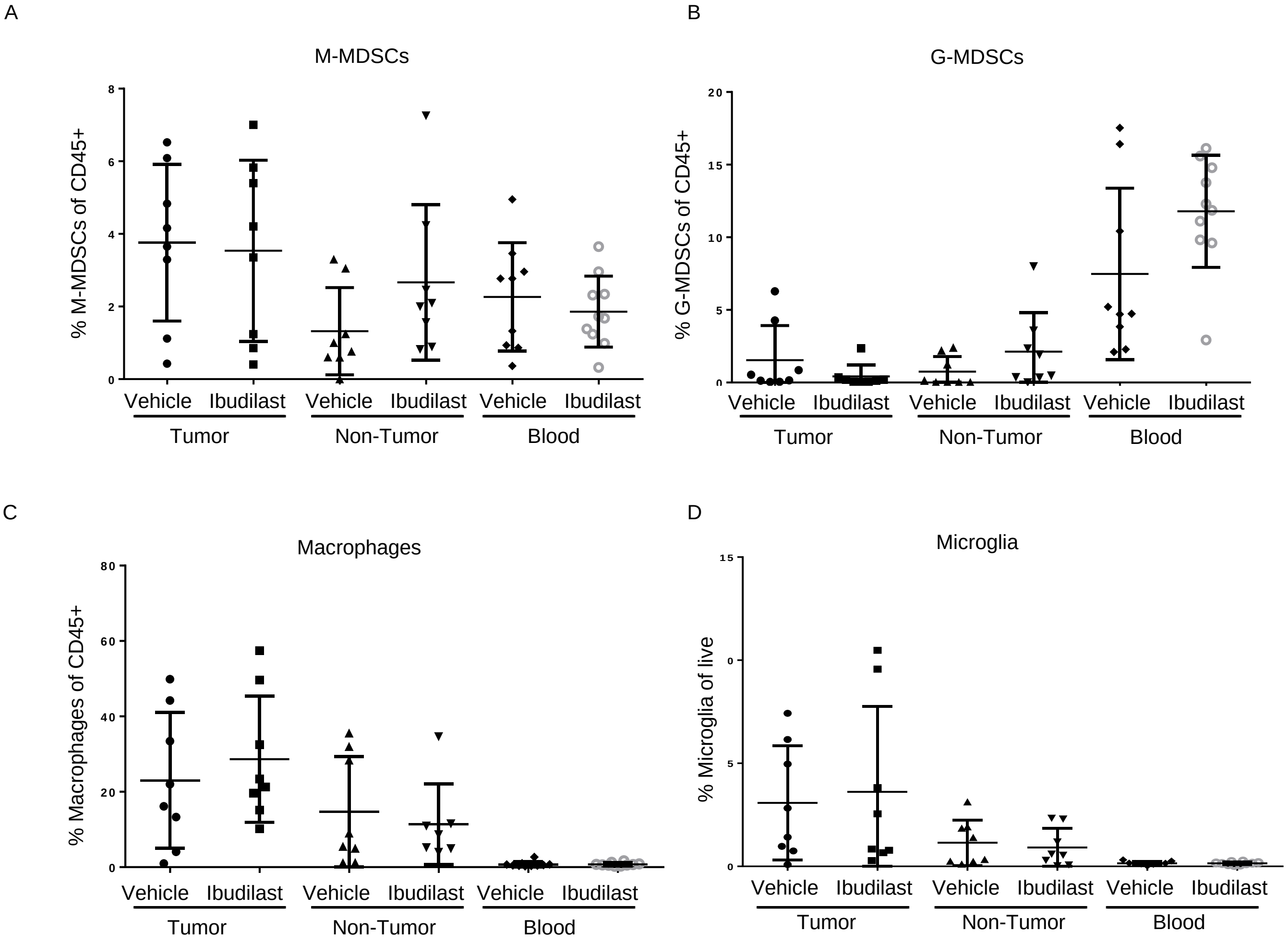

Supplemental Figure 3.

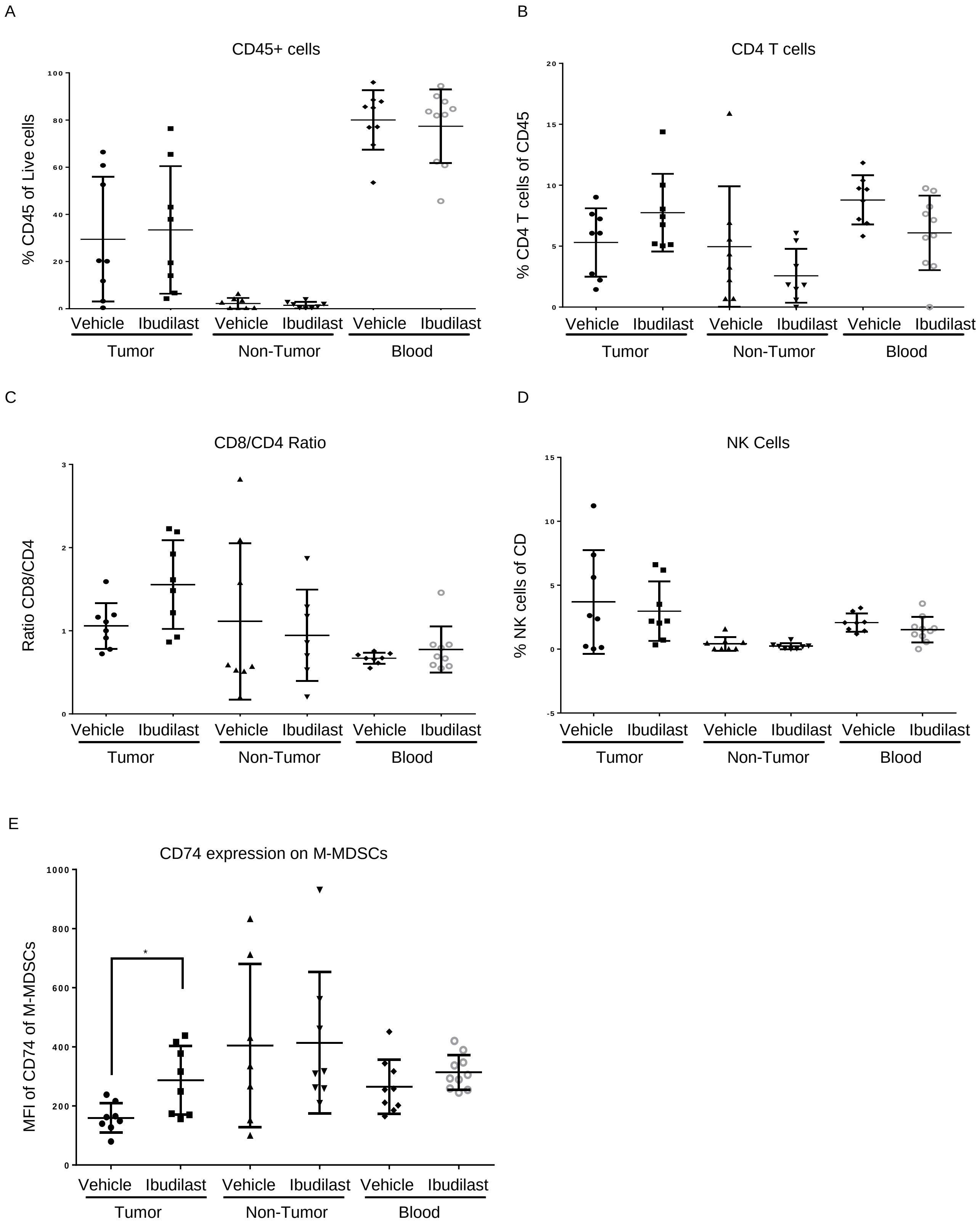

Supplemental Figure 4.

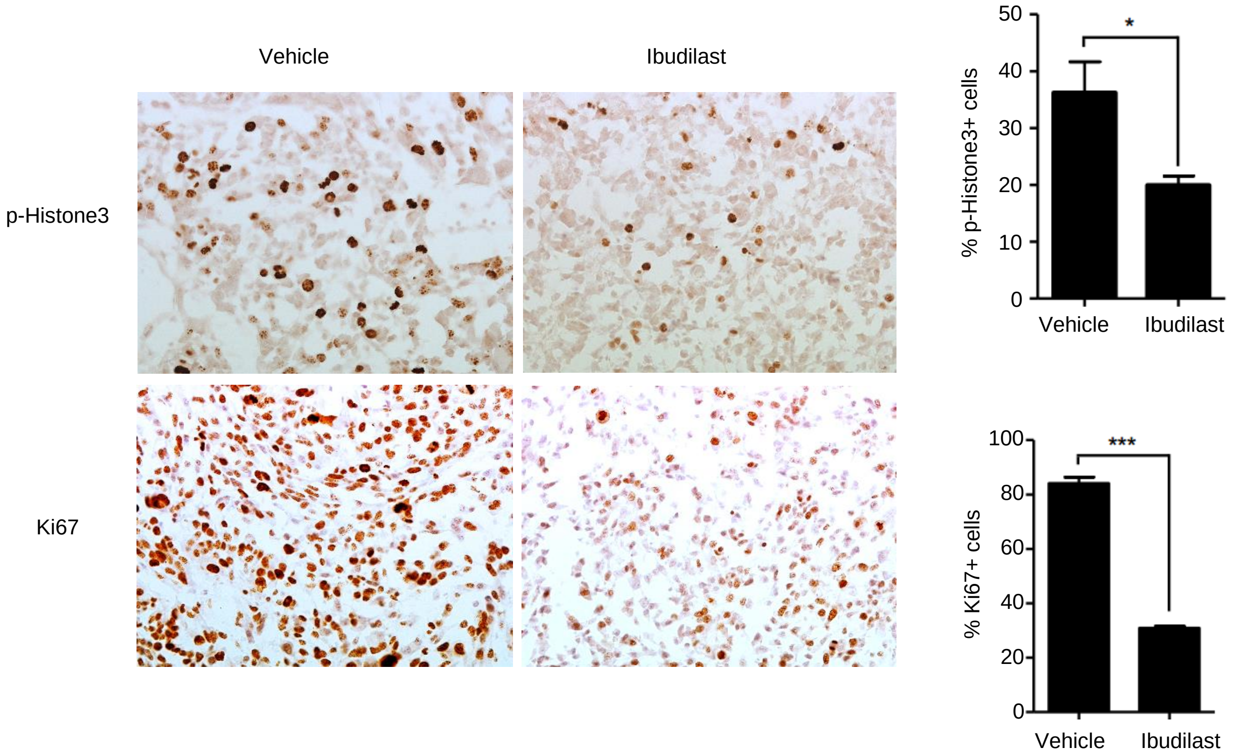

Supplement: Supplemental Figure 1 — shRNA knockdown of MIF in GL261 was performed using 2 separate shRNA's which were the top targets from previously published work from our group to generate stable knockdown cell lines of GL261 (A). Comparing the survival of intracranially implanted tumors in NSG immune incompetent mice demonstrate no survival difference in NSG mice (B). Treating n = 10 GL261 tumor bearing mice 2x weekly with anti-MIF antibody (gifted from Dr. Richard Bucala) vs. n = 10 IgG control treated mice demonstrated no survival benefit (C). MDSC co-culture dynamics over time analyzing n = 3 mice in separate co-cultures where one well was used each day over 7 days to check the number of CD45+ cells by flow cytometry (D). Survival curve analysis was performed in GraphPad Prism using Log-rank (Mantel-Cox) test for p value and hazard ratio log rank was computed on the same data using GraphPad Prism. [file Data_Sheet_1.pdf]
